# A review of coral reef restoration initiatives in the Western Indian Ocean Region

**DOI:** 10.1371/journal.pone.0348015

**Published:** 2026-05-08

**Authors:** Juliet Furaha Karisa, Omar Farouk, Nadeem Nazurally, Luca Saponari, Joshua Wambugu, Joan Kawaka, Gildas Todinanahary, Anne Laudisoit, Lorna Slade, Nancy Ogega, David Ouma, Edward Senkondo, Masanja Joram, Hassan Yussuf, Joshua Oginda, Margaux Hein, Caitlin Lustic, Tali Vardi, Phanor Hernando Montoya Maya, Jessica Levy, Kristen Maize, Baruch Rinkevich, Arthur Tuda, George Maina

**Affiliations:** 1 Kenya Marine and Fisheries Research Institute, Mombasa, Kenya; 2 REEFolution Trust, Diani, Kenya; 3 Department of Agricultural and Food Science, Faculty of Agriculture, University of Mauritius, Réduit, Mauritius; 4 Faculty of Fisheries, Hokkaido University, Hokkaido, Japan; 5 The Centre for Environment and Education, Nature Seychelles, Mahe, Republic of Seychelles; 6 Marine Animal Ecology and Environmental Policy Group, Wageningen University and Research, AH Wageningen, The Netherlands; 7 Coastal Oceans Research and Development in the Indian Ocean East Africa, Mombasa, Kenya; 8 The Nature Conservancy, Africa Regional Office, Nairobi, Kenya; 9 Institut Halieutique et des Sciences Marines, University of Toliara, Toliara, Madagascar; 10 Mwambao Coastal Community Network, Zanzibar, Tanzania; 11 Capacities for Biodiversity and Sustainable Development, Brussels, Belgium; 12 The Nature Conservancy, Arusha, Tanzania; 13 Northern Rangelands Trust - Coast, Lamu, Kenya; 14 MER Research and Consulting, Saratoga Springs New York, United States of America; 15 The Nature Conservancy, Arlington, Virginia, United States of America; 16 Coral Restoration Consortium, Tavernier, Florida, United States of America; 17 Israel Oceanography and Limnological Research, National Institute of Oceanography, Haifa, Israel; 18 Western Indian Ocean Marine Science Association, Zanzibar, Tanzania; Bigelow Laboratory for Ocean Sciences, UNITED STATES OF AMERICA

## Abstract

Widespread coral reef degradation in the Western Indian Ocean (WIO) underscores the need to support restoration to accelerate recovery in severely impacted areas. Restoration science in the WIO remains nascent, with few small-scale initiatives and limited integration between reef managers, practitioners, and researchers, hindering effective coordination of efforts toward larger-scale action. This study aimed to consolidate knowledge on WIO coral restoration initiatives, identify lessons learned, and assess current practices to inform future coordination and scaling efforts. We focused on (i) approaches and techniques used, (ii) lessons from successes and failures, and (iii) the potential role of a regional practitioner network in addressing identified gaps. Data were compiled from peer-reviewed literature, online sources, a WIO practitioner survey, and a regional workshop. Results indicate growing momentum for coral reef restoration, particularly in Kenya, Tanzania, and the Seychelles, with an increase in publications since 2021. Twenty-two active initiatives were identified across eight countries; 76% involved local communities, and 27% were fully community-led. Asexual methods like coral gardening dominated due to cost-effectiveness, while sexual propagation was limited to Seychelles and planned for Mauritius. Initiatives primarily targeted fast-growing genera such as *Acropora* and *Pocillopora*, with limited species diversity. Monitoring practices were highly variable, with most projects relying on short-term ecological indicators and few reporting standardized quantitative metrics such as survival rates or restoration footprint. While 86% of projects collected ecological baseline data, only 38% included socio-economic indicators. The synthesis of findings contributed to the establishment of the Western Indian Ocean Coral Reef Restoration Network (WIOCRRN), a regional platform guided by the Capacity, Access, Research, and Enhancement (“CARE”) framework, aimed at advancing resilient and sustainable reef ecosystems while aligning conservation outcomes with the socio-economic needs of coastal communities. The uneven geographic distribution of initiatives and documented data gaps highlight opportunities for improved coordination, standardization, and strategic scaling of restoration efforts across the region.

## 1.0. Introduction

The Western Indian Ocean (WIO) region; encompassing Kenya, Comoros, Seychelles, Mauritius, Madagascar, Tanzania, Mozambique, Somalia, South Africa, Mayotte, and Réunion, is home to approximately 10% of the world’s coral reefs and is recognized as a global biodiversity hotspot, hosting one of the highest concentrations of coral species outside the Coral Triangle [1]. These reefs contribute significantly to national economies, with millions of people in coastal communities depending on them for fisheries, tourism, and coastal protection [1,2]. Coral reefs in the WIO have experienced widespread degradation driven by recurrent marine heatwaves, coastal development, overfishing, and declining water quality, with recent assessments reporting substantial losses in live coral cover across multiple countries [1,3,4]. Despite their ecological and socio-economic importance, restoration and recovery efforts in the region remain limited in scale and unevenly documented, particularly when compared to more intensively studied regions such as the Caribbean and Pacific [5].

Historically, marine conservation in the WIO and globally has emphasized proactive measures, such as establishing Marine Protected Areas (MPAs) and improving water quality, to protect reefs and support natural regeneration. While traditional reef conservation has emphasized protection through marine protected areas, global syntheses increasingly recognize the need for active restoration in reefs that have crossed ecological thresholds and show limited natural recovery [6–8].

The UN Decade on Ecosystem Restoration (2021–2030) has placed ecosystem recovery, including coral reefs, high on the global agenda, aiming to scale up restoration to achieve Sustainable Development Goals (SDGs) linked to biodiversity conservation, poverty alleviation, improved livelihoods, food security, and climate action [4]. Globally, coral reef restoration has expanded rapidly over the past decade, yet outcomes remain highly variable due to differences in ecological context, methodological choices, monitoring frameworks, and long-term funding commitments [8,9]. Coral reef restoration is now widely recognized as a potentially powerful tool, particularly at local scales and on reefs with limited natural recruitment, when implemented alongside proactive conservation measures [10,11]. While not a silver bullet for reef decline, restoration can deliver ecological and socio-economic benefits, such as improved fish habitat, increased tourism opportunities, enhanced marine education and stewardship, and alternative livelihoods [12,13].

In the WIO, restoration activities have been documented since at least the early 2000s, but have remained scattered and often small-scale. Although notable projects exist, particularly in Kenya and Seychelles [14–17], many efforts remain undocumented in the scientific literature. A lack of communication, coordination, and knowledge-sharing between managers, practitioners, and researchers has hindered the synthesis of lessons learnt, increased the risk of duplicating trials, and limited opportunities to optimize techniques for local conditions [8].

This manuscript addresses these gaps by synthesizing available information on past and ongoing active coral reef restoration initiatives in the WIO region. Our three primary aims are to: (i) review the approaches and techniques currently used, (ii) identify key lessons learned from both successes and failures, and (iii) explore the potential for establishing a WIO Coral Reef Restoration Network to improve coordination and impact. Through this analysis, we aim to support the development of collective, science-based restoration efforts that enhance reef resilience and sustain the ecosystem services on which WIO communities depend.

## 2.0. Methodology

We compiled information on coral reef restoration activities in the Western Indian Ocean (WIO) region using three complementary approaches (adapted from [8] and [18]): (i) a structured literature review, (ii) an online practitioner questionnaire, and (iii) a regional stakeholder workshop. Each component is described below in the order presented. Data on existing restoration programs were classified by: (1) geographical location, (2) source of information, (3) methods used, (4) stakeholders involved, (5) monitoring approaches and indicators of success, and (6) lessons learned, challenges, and opportunities.

We included only reactive coral restoration projects, defined as active interventions to directly enhance coral survival or regrowth (e.g., transplantation, nursery propagation, predator removal, algal removal). Proactive conservation interventions, such as Marine Protected Areas, water quality management, or projects without coral-specific components, were excluded because their primary aim is to protect or improve environmental conditions rather than directly restore coral cover. This decision allowed us to focus the review on projects involving direct restoration actions, which are more readily comparable in terms of methods and outcomes. However, this exclusion means the synthesis does not capture the full spectrum of activities contributing to reef recovery in the WIO, and we acknowledge that proactive measures can strongly influence the context and success of reactive interventions.

As regards questionnaire bias, we recognised potential underreporting of restoration efforts and possible site overlap in questionnaire responses, but we did not attempt to correct for or estimate the magnitude of this bias. These limitations are considered in the discussion, including how such bias might influence the apparent geographic distribution of projects (e.g., Kenya’s apparent dominance may partly reflect stronger documentation networks).

Following method in [8], we avoided interpretative bias except when identifying key priorities for the development and implementation of coral restoration in the WIO. These priorities, based on practitioner input, are presented later in the results (section 3.2.10) and discussed in section 4.0.

### 2.1. Literature reviews

We combined information from i). peer-reviewed literature, and ii). Gray literature, including online archives.

#### 2.1.1. Peer-reviewed literature.

To obtain the most comprehensive coverage of the scientific literature, we conducted a systematic review of peer-reviewed articles in two widely used databases: Scopus and Google Scholar. We used a combination of search terms associated with coral reef restoration in the WIO region to ensure complete coverage.

First, we searched Google Scholar using the keywords “coral + restoration + [country name]” for each WIO country. This search included an exclusion criterion for year of publication; only articles from 1990 to 2024 were considered to capture restoration trends over the past four decades. This search, conducted on 12 November 2024, yielded 60 articles.

We then conducted a complementary Scopus search using the following query:

*TITLE-ABS-KEY (coral AND restoration AND ‘country name’) AND PUBYEAR > 1989 AND PUBYEAR < 2024* for all 11 WIO countries. The Scopus search was run on 15 November 2024 and returned 48 articles.

Titles and abstracts were manually screened, duplicates removed, and only studies fulfilling the criteria for reactive coral restoration approaches were retained. The final dataset comprised 24 articles. For each study, we recorded the title, authors, year of publication, restoration technique, results, and lessons learned in an MS Excel spreadsheet.

#### 2.1.2. Grey literature and online archives.

Because many WIO restoration projects operate without formal scientific involvement, we also reviewed non-peer-reviewed sources. We searched Google (for general web content), Google Scholar (for unpublished reports), and YouTube (for visual project documentation) using the search term “coral restoration + [country name].”

To improve reliability, we verified grey literature findings by triangulating information where possible, for example, by cross-checking project details across multiple independent sources (e.g., a project website and a news report, or a video and an NGO’s annual report). Only project details that could be confirmed through at least two sources, or that came from a known, credible organization, were retained.

From these sources, we recorded only basic, verifiable information: the project location, methods used, and reported outcomes or lessons learned. We acknowledge that, despite these verification steps, grey literature inherent has reliability limitations, which may affect completeness and accuracy.

### 2.2. Online questionnaire

We developed an online questionnaire targeting coral reef restoration practitioners in the WIO region to capture information unavailable in published sources and to build upon the findings from the literature review. The objectives were to: (i) document ongoing and past restoration initiatives and lessons learned, and (ii) gather information to inform the potential formation of a WIO reef restoration network. All participants provided informed consent for their data to be used in this publication.

The questionnaire was administered in two stages. In the first stage, approximately 15 participants from Kenya were invited to a virtual meeting on May/June 2024, during which they participated in small-group discussions (breakout rooms) based on the specific restoration projects they were working on. These discussions served as a focus group exercise, and participants completed an online questionnaire (33 questions; format adapted from [8]). A total of seven completed questionnaires were collected in this stage. In the second stage, a revised questionnaire (30 questions, including items on the potential for a regional restoration network) was sent to 18 practitioners from Tanzania, Seychelles, Mauritius, Madagascar, Comoros, Mayotte, and Mozambique in June/July 2024. Practitioners were identified via professional networks (including the 12^th^ WIOMSA Scientific Symposium) and online searches. Fourteen responses were received. Participants were instructed to submit a separate questionnaire for each restoration site; however, some may have combined multiple sites into a single response, potentially underestimating the total number of sites represented.

The questionnaire (provided as S1 Appendix) included 27 optional questions, allowing respondents to skip items they preferred not to answer. We acknowledge that the questionnaire findings may present a biased view of the number and distribution of restoration sites in the region. In addition, the questionnaire was not sent to practitioners in some WIO countries (e.g., Somalia, South Africa, Réunion) where the authors were unaware of any restoration activities at the time of the study. It is possible that undocumented restoration is occurring in these countries, and therefore, the dataset may be incomplete. Because participation was voluntary and recruitment relied on existing professional networks, the dataset likely reflects a bias toward more active, visible, or externally supported initiatives.

### 2.3. In-person workshop

An in-person workshop was held in Zanzibar, Tanzania, from July 16^th^ to 18^th^ 2024, to which representatives of multiple coral restoration projects from the WIO region were invited. The workshop aimed to generate a network of reef restoration practitioners to facilitate the growth of coral restoration initiatives in the region as a solid, resilient, and scientifically rigorous practice.

### 2.4. Data analysis

We conducted a qualitative synthesis using summary statistics (counts and percentages) to compare restoration project characteristics and outcomes, following method in [8]). Projects missing data for a given question were excluded from analysis for that question. For multi-select questions, each selected option was counted separately, meaning total responses could exceed the number of projects. Percentages were calculated from the proportions of projects/sites that responded to each item. In addition to descriptive statistics, we explored potential patterns in restoration outcomes across projects. However, due to the limited sample size, heterogeneity of project characteristics, and incomplete reporting across variables, formal comparative analyses (e.g., cross-tabulations) were not undertaken. The analysis, therefore, remained descriptive, focusing on summarizing patterns in restoration approaches, monitoring practices, and practitioner-reported outcomes. Survey responses were aggregated using frequencies and proportions, and qualitative responses were thematically grouped to identify recurring challenges and lessons learned. No inferential statistical analyses were conducted due to the heterogeneity of projects and the non-random nature of the dataset.

### 2.5. Mapping of information gathered

Mapping was carried out for restoration projects that provided site information or mentioned countries whose locations could be identified using Google Maps. While some data included exact GPS coordinates, others only referenced place names. In such cases, these names were used as proxies to approximate the locations of restoration activities within the WIO region.

### 2.6. Limitations

This study is subject to several limitations. First, the survey relied on self-reported information, which may introduce reporting bias or inconsistencies in terminology (e.g., project scale or technique categorization). Second, some projects lacked complete quantitative monitoring data, limiting comparative analysis across initiatives. Third, while both peer-reviewed and grey literature were reviewed, unpublished projects may not have been captured. Finally, most projects were short-term (<5 years), restricting assessment of long-term ecological outcomes and resilience to large-scale disturbances.

### 2.7. Ethics statement

This study did not involve experimental manipulation, clinical trials, or the collection of sensitive personal data. The online questionnaire and workshop targeted professionals acting in their institutional or organizational capacity, and participation was entirely voluntary and anonymous. No personal identifiers were collected. Based on institutional guidance, this study was therefore exempt from formal ethical review. Written confirmation of this exemption has been provided as supplementary documentation. The protocol for this study was reviewed and approved in accordance with the ethical standards and guidelines of the Kenya Marine and Fisheries Research Institute. All participants provided freely given, informed written consent prior to taking part in the questionnaire.

## 3.0. Results

### 3.1. Literature review

The number of scholarly publications on coral reef restoration in the WIO has increased steadily over recent decades, indicating a growing focus on restoration efforts in the region. Between 1990 and 2024, publications output rose gradually, with a notable uptick beginning in 2021. In the early years, an average of one publication was produced annually, while from 2021 to 2024, as many as three publications appeared each year. Although we did not calculate an exact fold-change, this represents a substantial increase compared to the early 2000s. Kenya recorded the highest number of publications (n = 9), followed by Seychelles (n = 7).

The most common topics addressed in the literature include the establishment and optimisation of coral nurseries, species-specific propagation techniques, and the ecological impacts of restoration interventions. Some previous studies focused mainly on propagation techniques tailored to specific coral taxa [12,17,19]. Other research has assessed the ecological outcomes of restoration, identifying both positive and negative effects of interventions (e.g., [17,20]). Studies on artificial reef structures and transplantation methods (e.g., [16,17]) demonstrated potential benefits for coral cover and biodiversity. Several publications also highlight community-based approaches, underscoring the socio-economic benefits of restoration alongside ecological gains, particularly for coastal communities reliant on reef ecosystems.

Three primary restoration techniques emerged from the literature review, with distinct frequencies of use:

i) Coral gardening (63% of documented efforts), defined here as the cultivation of coral fragments in nurseries, either in situ or ex situ, until they reach a size suitable for transplantation to degraded reef sites [21].
ii) Direct coral transplantation (30%), in which corals are moved directly from donor to recipient sites.
iii) Substrate creation (7%), involving the deployment of artificial structures to facilitate coral recruitment and growth.

Grey literature search identified only limited region-specific documentation meeting the inclusion criteria. Most documents lacked methodological detail or quantitative outcomes and were therefore not included in a detailed synthesis.

### 3.2. Online questionnaire

#### 3.2.1. Restoration context.

**Fig 1:**
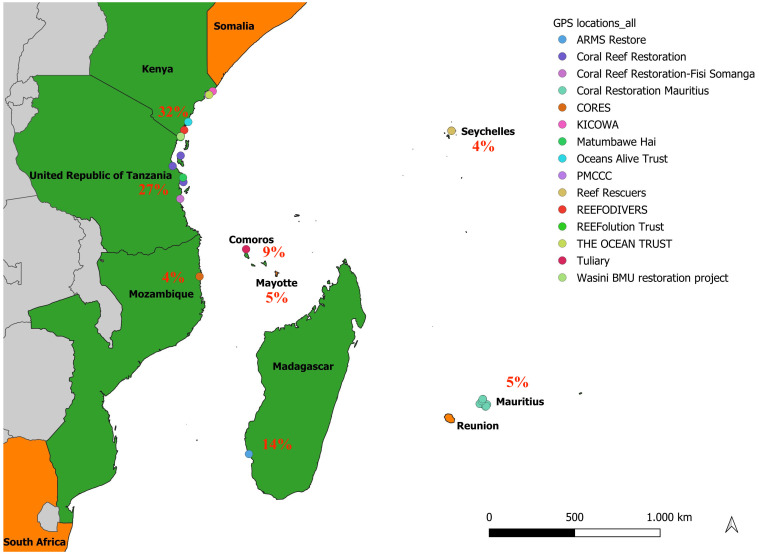
Spatial distribution of coral reef restoration initiatives across the Western Indian Ocean (WIO). Circles indicate the locations of each shared restoration point. Colors of each circle indicate the different projects; corresponding project names and GPS coordinates and countries are provided in S1 and S2 Tables. Countries in colors (green and orange) mark the full stretch of the WIO region, while countries in green indicate the 8 of 11 WIO countries with active coral restoration projects. *Base map data were obtained from Natural Earth and OpenStreetMap and created using QGIS. All map data is publicly available and licensed for reuse under terms compatible with Creative Commons Attribution 4.0 International (CC BY 4.0) license; no third-party or proprietary map data was used*.

The study identified 22 coral restoration sites across 8 of the 11 WIO countries, including Kenya, Tanzania, Madagascar, Comoros, Mozambique, Seychelles, Mauritius, and Mayotte. Fifteen of these projects provided locations. A map of the WIO region highlighting countries with active coral restoration projects is shown in **Fig 1**. Restoration sites were mostly concentrated in Kenya (32%) and Tanzania (27%). Non-Governmental Organizations (NGOs) supported 35% of the restoration activities, followed by Community-Based Organizations (CBOs) at 27%. In total, 22 active restoration sites were identified, implemented by 19 distinct organizations across the WIO.

**Fig 2:**
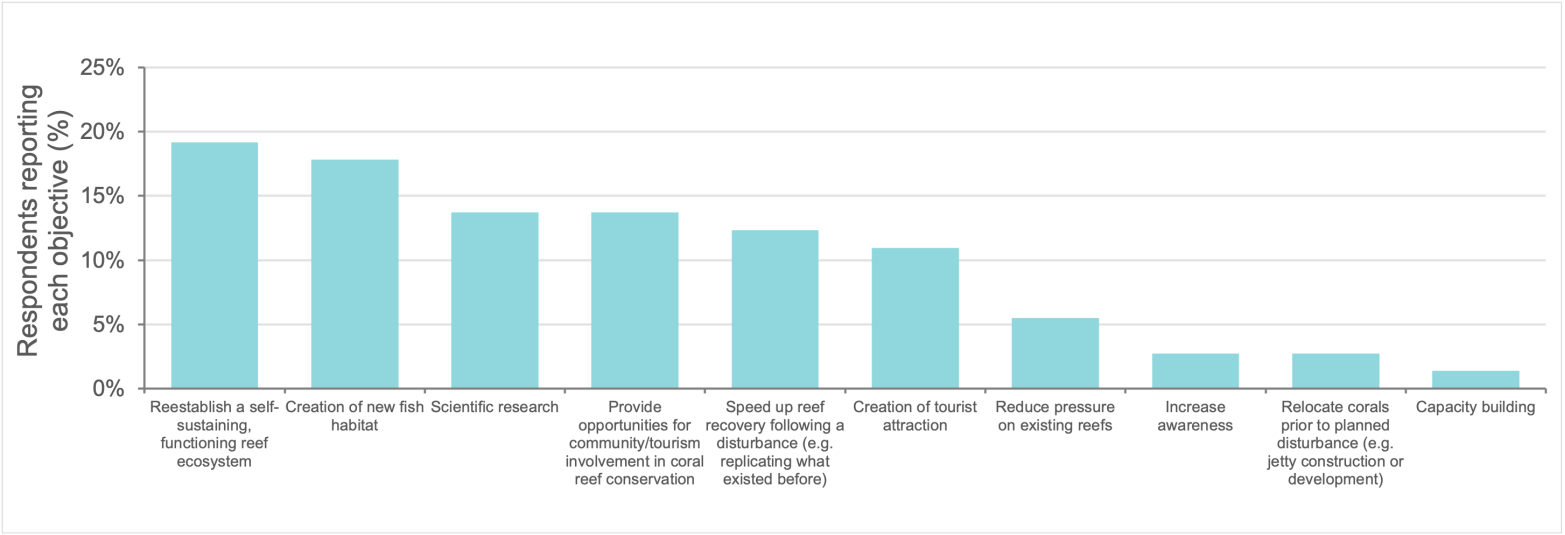
Distribution of coral reef restoration approaches reported by surveyed initiatives in the Western Indian Ocean. Values indicate the proportion of initiatives that apply each approach; multiple approaches could be reported by a single project (n = 22 projects).

The restoration goals reported by respondents included reestablishing a self-sustaining, functioning reef ecosystem (19%), creating new fish habitat (18%), conducting scientific research (14%), and providing opportunities for community and tourism involvement (14%) (**Fig 2**). Note that respondents had the option to select multiple goals. Restoration initiatives across the Western Indian Ocean were implemented using a limited number of dominant restoration techniques. Most projects relied on asexual methods, particularly coral gardening and direct transplantation, reflecting their relatively low cost and logistical feasibility. In contrast, sexual propagation approaches were rare and confined to a small number of initiatives, primarily in the Seychelles, with pilot or planned applications reported elsewhere in the region.

#### 3.2.2. Level of community involvement.

Local communities participated in 76% of coral reef restoration activities across the 22 recorded sites. For this study, “local communities” broadly include local residents, fisher groups, community-based organizations (CBOs), and Beach Management Units (BMUs), though the extent of involvement varied. It remains unclear whether community participation covered the entire restoration process, from planning and implementation to monitoring, or was limited to specific stages such as awareness campaigns, the build-up of coral restoration frames (e.g., constructing nursery structures like spider frames), capacity building on coral reef restoration, coral out-planting, and monitoring activities. Of these initiatives, 27% were fully community-led, while an additional proportion involved communities as implementation partners. Community involvement was a prominent feature of restoration initiatives across the region. Most projects reported engaging local stakeholders through activities such as site maintenance, coral nursery operations, and awareness programs, while a smaller subset of initiatives were fully community-led, with local groups responsible for decision-making and implementation. S3 Table provides a detailed breakdown of the activities in which local communities were involved during the restoration process.

#### 3.2.3. Types of corals and restoration techniques.

**Fig 3:**
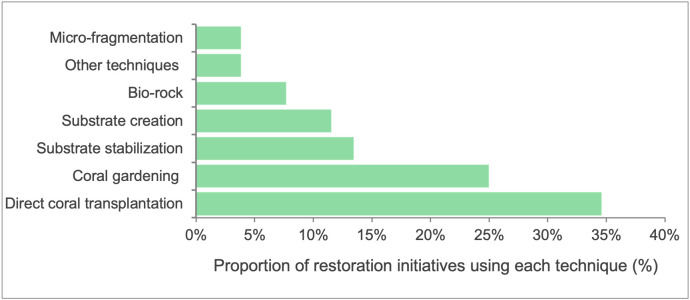
Distribution of coral reef restoration techniques reported across surveyed initiatives in the Western Indian Ocean. Percentages represent the proportion of survey respondents selecting each objective (n = 22 responses; multiple responses permitted).

A total of seven distinct coral restoration techniques were recorded through the surveys. This contrasts with previous reports in the literature, which indicated that a larger proportion of sites (e.g., 63%) employed specific restoration methods. Direct transplantation was the most frequently reported technique, accounting for 35% of the restoration projects (**Fig 3**). This method involves moving coral fragments or colonies directly from a donor site to the restoration site, unlike coral gardening, which first grows orals in nurseries before outplanting. Coral gardening was employed in 25% of the projects, while 13% utilized substrate stabilization methods. The addition or creation of substratum, such as artificial reefs, was reported in 12% of the projects. “Substrate stabilization” refers to interventions aimed at securing existing loose reef material (e.g., rubble consolidation using mesh, frames, or tie-wrap systems) to enhance natural recruitment. In contrast, “substrate addition or creation” involves introducing new artificial or natural materials (e.g., concrete modules, reef balls, limestone structures) to increase available settlement surface where structural complexity has been severely reduced.

“Biorock” techniques were applied in 8% of the restoration projects, and micro-fragmentation was used in less than 4%. Other innovative methods were also trialled at some sites. For instance, Autonomous Reef Monitoring Structures (ARMS) were used at 4% of the locations surveyed. While ARMS is primarily a monitoring tool designed to assess settlement and recruitment of fauna, in certain projects, it was incorporated to evaluate the ecological outcomes of restoration efforts. In some cases, multiple techniques were combined at a single site.

**Fig 4:**
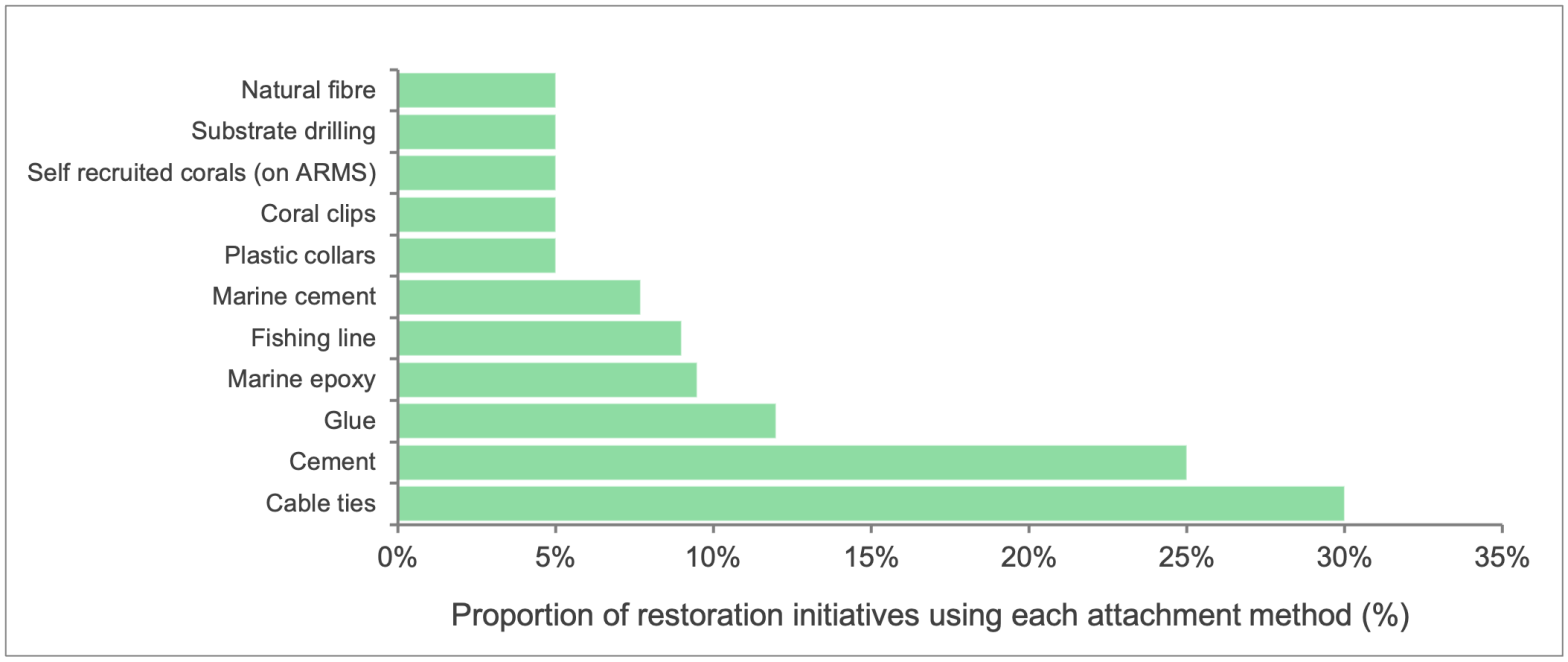
Percentage occurrence of methods used for attaching coral fragments across restoration sites in the WIO region, showing the relative frequency of each technique (n = 22 responses; multiple techniques per project possible).

Attachment methods varied across projects, with cable ties being the most commonly used technique (29%; **Fig 4**), followed by cement (22%). Several initiatives reported using multiple attachment methods, while others relied on a single approach.

**Fig 5:**
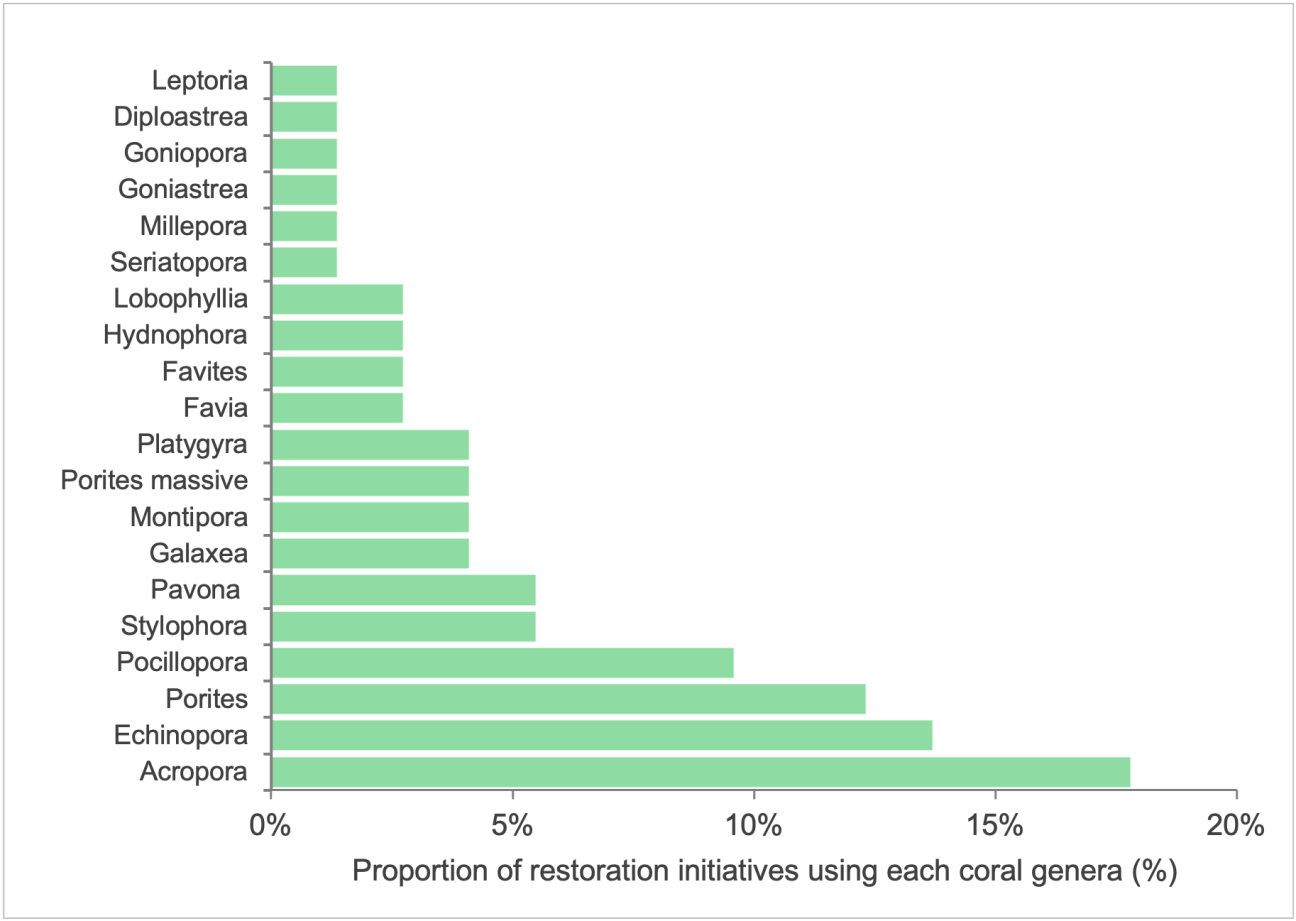
Coral genera used in restoration projects across the Western Indian Ocean (WIO), highlighting the predominance of Acropora and the range of genera transplanted.

Coral taxonomy and morphology were also recorded, revealing that all restoration activities involved multiple coral taxa. The majority of restoration projects focused on fast-growing branching corals, which accounted for 42% of the projects (**Fig 5**). A total of 20 coral genera were identified, with a diverse range of species employed in restoration activities. However, as respondents did not provide species-level identification, only genera are represented in the results. The genus *Acropora* was used in 18% of the restoration projects.

#### 3.2.4. Size of restoration area and number of years since restoration activity began.

**Fig 6:**
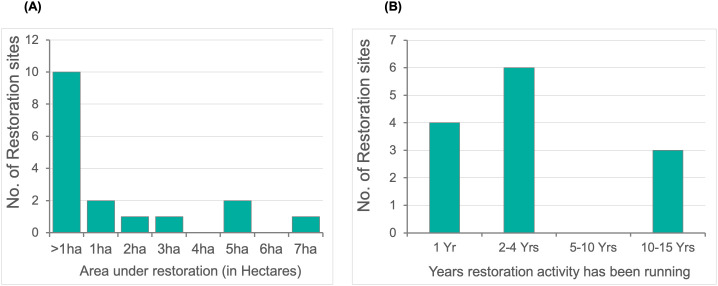
(a) Size of coral reef restoration sites in the WIO region. (b) Number of years since the inception of restoration activities at a site (n  =  22 projects).

Most restoration areas were small, with 63% of restoration sites covering less than 1 Ha (**Fig 6a**). There was initially a report of a large-scale coral reef restoration site in the Seychelles covering 25 Ha; however, this has been corrected following verification.

Coral restoration initiatives are ongoing in the region, but they are dominated by short-term initiatives. Only 3 of the projects have been in existence for more than 10 years, with the oldest restoration site having been active for 15 years, indicating that most restoration initiatives in the region are relatively recent (**Fig 6b**).

#### 3.2.5. Collection of baseline information and measures of success.

Most respondents (86%) reported collecting baseline information before beginning restoration activities at a site. However, the methodologies used to conduct baseline surveys varied between respondents. Most of the baseline surveys collected ecological information and only 38% of the initiatives collected baseline information for the socio-economic components.

Monitoring practices varied widely among initiatives. Most projects focused on short-term ecological indicators such as coral survival and visual assessments of colony condition, while fewer initiatives incorporated longer-term indicators, related to reef structural complexity or socio-economic outcomes. The absence of standardized monitoring frameworks limited direct comparison across sites.

Quantitative metrics such as the number of coral fragments outplanted, survival rates, and total restoration footprint were inconsistently reported across initiatives and were therefore not comparable at the regional scale. Most projects relied on short-term ecological indicators, including visual assessments of coral condition or presence–absence observations, rather than standardized quantitative measures of restoration performance.

#### 3.2.6. Achievements, challenges, and solutions.

Around 43% of respondents reported that their restoration projects were successful, 19% partially successful, and only 5% reported a negative outcome from restoration activities. The remaining 23% did not answer this question. Respondents highlighted a range of notable achievements, which, when grouped thematically, reflect advances in restoration methods, ecological recovery, capacity building, community engagement, visibility, and financial support. These include the introduction of innovative technologies and improved techniques (e.g., ARMS in Madagascar, strategic site selection), strengthened local capacity and education (including youth and women’s empowerment), measurable ecological gains such as increased coral cover, fish diversity, and recruitment, deepened community stewardship, greater visibility through local and international recognition, and the attraction of sustained donor and institutional support. Selected respondent quotes are presented in **Table 1** illustrates these categories.

**Table 1:** Reported outcomes and notable achievements of coral reef restoration projects across the WIO, categorized with selected respondent quotes.

Category	Description	Illustrative Quotes/ Examples of reported achievements
1. Technological innovation & improved techniques	Adoption of advanced methods and careful site selection to improve restoration success.	“Use of ARMS in Madagascar has enhanced biodiversity monitoring.” “Choosing good relocation sites and methods has been key.”
2. Capacity building & education	Training and involvement of students, community members, and rangers; empowerment of women and youth.	“Six local communities trained, four now employed in restoration organizations.” “Promotion of women in fisheries through *Mama Matumbawe* and *Mama Pweza* initiatives.”
3. Ecological gains	Increased coral cover, fish diversity, and natural recruitment.	“Significant increase in fish diversity and coral recruitment.” “Over 1,000 coral fragments planted, with strong survival rates.”
4. Community engagement & stewardship	Strengthened links between people and environment; sustained local involvement over many years.	“Enhanced community stewardship with restoration ongoing for 11 years.” “From non-swimmers to advanced restoration divers—people now lead conservation locally.”
5. Visibility & recognition	Local and international exposure through media, awards, and visits.	“Featured in documentaries and news stories, sparking new conservation initiatives.” His Royal Highness King Charles III visit (2023).” “Recipient of UNDP Equator Prize (2017).”
6. Financial & institutional support	Attraction of donor funding and institutional partnerships.	“Increased donor support from multiple organizations.” “Prestigious awards helped secure further funding.”

**Fig 7:**
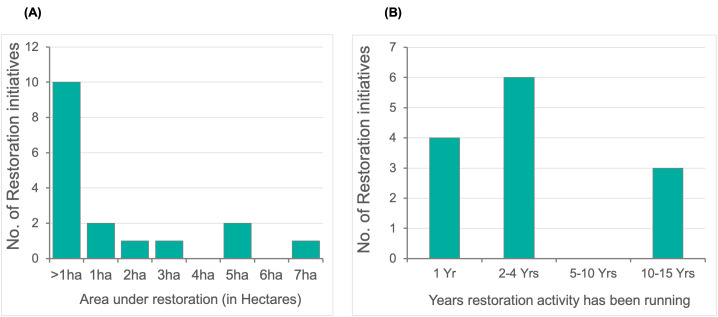
Thematic grouping of challenges reported by coral reef restoration initiatives in the Western Indian Ocean. Percentages represent the relative frequency of challenge themes reported by respondents across all initiatives (n = 49 total reports).

The challenges faced by respondents in coral restoration activities were grouped into nine thematic areas. Broadly, these revolved around: (1) insufficient funding, (2) difficulties with procurement of necessary materials (e.g., epoxy not locally available) and logistical delays, (3) unfavourable environmental conditions, (4) poor management, (5) lack of expertise and capacity in coral reef restoration, (6) weak linkage of restoration initiatives to human and social dimensions, (7) lack of sustainable measures, (8) threats to transplanted corals from climate change, and (9) low levels of awareness (**Fig 7**). Lack of sustainable measures refers to the absence of long-term funding mechanisms, institutional support, and post-project monitoring plans. A complete list of reported challenges is provided in **Table 2**.

**Table 2:** Thematic categories of challenges reported by coral reef restoration initiatives in the Western Indian Ocean. Numbers and percentages represent the frequency of reports across surveyed initiatives (n = 49 total reports). Illustrative examples summarize the types of challenges described by practitioners.

Thematic category of challenges	Number of reports (n)	Percen-tage (%)	Illustrative examples of reported challenges
Procurement and logistics	8	16	Limited access to boats, engines, diving gear, and underwater equipment; delays due to procurement procedures; high logistical costs; working underwater with minimal infrastructure
Funding	8	16	Lack of long-term programmatic funding; mismatch between funder timelines and on-the-ground realities; high operational costs; insufficient resources to implement planned activities
Ecological/ physical conditions	7	14	Crown-of-thorns starfish outbreaks; coral predation by Drupella and Coralliophila snails; rough seas and strong currents; sedimentation; poor matching of site conditions with restoration methods
Management	6	12	Lack of political support; ongoing fishing activity damaging outplanted corals and structures; weak site management and enforcement; absence of formal site designation
Expertise/ capacity	6	12	Lack of skilled local personnel; limited technical expertise for long-term planning and monitoring; first-time implementation with no regional reference projects; difficulty harmonizing scientific and community-based data
Human/ social	5	10	Limited community acceptance of restoration as a new activity; informal or absent MOUs with communities; conflicts related to perceived financial benefits; slow ecological results, reducing community engagement
Post-project monitoring	4	8	Absence of formal monitoring plans; failure to implement monitoring effectively; limited capacity and resources for long-term follow-up
Climate change	3	6	Coral bleaching events linked to elevated sea temperatures; climate-induced stress reducing survival of outplanted fragments
Awareness	2	4	Limited community awareness of coral restoration objectives; continued extractive activities due to low awareness
Total	**49**	**100**	

Challenges reported by practitioners clustered into a limited number of recurring themes (Table 2). Financial constraints and limited long-term funding cycles were the most frequently cited barriers, followed by gaps in technical capacity and training. Institutional challenges, including limited government coordination and regulatory support, were also prominent. In contrast, climate change was mentioned less frequently than local operational constraints. Destructive fishing and insufficient enforcement were highlighted in several cases, particularly where restoration sites overlapped with subsistence fishing grounds.

Respondents suggested solutions corresponding to the challenges identified above. (1) To address funding limitations, participants emphasized stronger leadership, advocacy, and improved grant-writing capacity to secure longer-term financial support. (2) To overcome limited technical capacity and coordination gaps, the use of AI-enabled underwater camera systems was proposed to enhance environmental monitoring and facilitate information sharing among practitioners. (3) To strengthen stakeholder engagement and institutional support, respondents recommended more frequent meetings and clearer communication of project benefits to communities and government agencies. (4) To mitigate procurement and logistical delays, participants suggested requesting project extensions and maintaining continuous monitoring to avoid data gaps. (5) No concrete solutions were proposed specifically for climate change impacts, highlighting a gap in climate-adaptive planning within current restoration initiatives.

#### 3.2.7. Key lessons learnt from coral reef restoration efforts.

The diverse array of lessons learnt reported by respondents were categorized into 11 thematic areas, reflecting insights on how coral reef restoration influences coastal communities, strengthens institutions, and advances ecological and scientific progress. The key lessons included: 1. climate change, especially coral bleaching, severely limits the success of traditional coral gardening, 2. restoration is costly, requiring significant and sustained funding, 3. large, institutionally supported projects with strong, transdisciplinary partnerships and stakeholder engagement are more likely to have long-term impact than smaller community-led initiatives, 4. meaningful community participation, from inception to implementation, especially involving women and youth, fosters ownership and project sustainability, 5. coral restoration is a long-term process, requiring patience to see ecological and social benefits, 6. regular monitoring and adaptive management are essential for tracking progress and improving outcomes, 7. awareness and capacity-building efforts increase local stewardship, 8. conservation and restoration should be integrated, 9. careful site selection, timing, and species choice are vital for ecological success (S4 Table).

#### 3.2.8. Opportunity and potential for a WIO reef restoration network.

Addressing the question of whether a WIO regional network of coral reef restoration practitioners would be of any value, 52% said yes, 5% maybe, 5% no and 38% did not respond.

### 3.3. In-person workshop

Forty-one participants from regional, national, and local governments and institutions, CSOs, and individual reef restoration practitioners to establish a Network: 1. Foster national, regional & global collaboration and connection between the network of practitioners and key stakeholders (virtually & in-person). 2. Build the network member’s capacity and skills surrounding reef restoration best practices. 3. Facilitate sharing and creation of knowledge, science, and coral reef interventions between practitioners in the WIO and globally, and 4. Advance supporting actions such as policy advocacy, monitoring and evaluation, funding opportunities, and career development for the network. The Network would provide a framework within which the various challenges experienced in advancing reef restoration would be addressed, and opportunities explored to realize impactful reef restoration in the WIO.

## 4.0. Discussion

### 4.1. General observations

#### 4.1.1. Regional expansion and scope of restoration.

This review summarizes the current status of coral restoration in the WIO, and to our knowledge, it is the most comprehensive review available in this area. In recent decades, scientific publication and the research-led approach have increased in the WIO region, especially since 2021. This trend underscores the growing recognition of coral reef degradation in the WIO and the urgency to address it through restoration and restoration science. The increase from nine case studies reported in [8] to the 22 documented here indicates that restoration practice is expanding geographically and institutionally across the region, including in relatively remote areas such as Comoros and Mayotte.

This highlights the significant contribution to coral reef restoration research and the active engagement in conservation efforts. However, the main focus of existing research has been limited to preliminary stages of restoration practices, such as initial assessment, nursery, and stocking. Only a few publications focused on transplantation methods, the use of artificial structures, the ecological impacts of restoration intervention, and the benefits to the local community.

Despite the progress, significant challenges persist. Many studies report variable success rates influenced by site-specific factors, species selection, and environmental conditions. Across the 24 peer-reviewed articles reviewed, success was consistently context-dependent, shaped by hydrodynamics, sedimentation, thermal stress exposure, and post-outplanting maintenance regimes. Lessons from these efforts point to the need for adaptive management strategies that integrate ecological monitoring with active restoration. Additionally, enhancing community engagement and ensuring cost-effective methodologies have been consistently identified as critical for scaling restoration efforts. The knowledge generated through these studies offers valuable insights into the restoration potential across the WIO. As restoration efforts evolve, an increasing focus is on aligning scientific methodologies with local socio-economic contexts to ensure ecological resilience and community empowerment. To note that across projects, ‘restoration success’ was most often interpreted as short-term fragment survival rather than recovery of reef structure, function, or self-sustaining coral populations, highlighting the need for clearer definitions of what constitutes a ‘restored’ reef at the site and regional scale.

#### 4.1.2. Restoration techniques and species selection.

Beyond describing restoration practices, the results identify key methodological drivers of success and failure in the WIO, particularly in relation to restoration techniques and species selection. The dominance of direct transplantation raises concerns about donor-site impacts where fragment extraction is not monitored. Species selection is strongly biased toward fast-growing branching corals (e.g., *Acropora*), which may limit long-term resilience under repeated thermal stress. In addition, the widespread use of cable ties, while practical and low-cost, may carry environmental trade-offs related to plastic persistence and maintenance requirements.

Restoration techniques and species vary widely across case studies, highlighting differences in goals, locally specific needs, site-specific environmental conditions, staff capacity, funding availability, and source of donor colonies. Additionally, all techniques mentioned are asexual and ocean-based. Assisted sexual reproduction (defined as the collection or production of ex-situ gametes, assisted lab fertilization, and settlement as per [22]) are not currently in use, although planning for a land-based nursery and the use of sexual reproduction are currently underway in the Seychelles (Nature Seychelles) and Mauritius (MOI – Mauritius Oceanographic Institute). Most case studies reported direct transplantation as the main restoration technique, followed by coral gardening (which includes the nursery step and use of iron structures, namely coral frames, as nursery grow-out structures). Direct transplantation involves moving coral fragments or colonies directly from a donor site to the restoration site, unlike coral gardening, where corals are first grown in nurseries before being outplanted.

Meanwhile, coral attachment methods vary according to the restoration stage and substrate type. Survey responses indicate that cable ties (tie-wraps) are the most frequently reported attachment method overall, particularly during nursery grow-out phases, whereas cement-based fixation is more commonly applied during final outplanting onto consolidated substrate. This distinction reconciles apparent methodological inconsistencies and reflects stage-specific attachment strategies. The stocking, nursery structure, and attachment methods reported in this study are relatively efficient and cost-effective depending on site-specific conditions [8]. Nevertheless, the widespread use of plastic cable ties raises environmental concerns, including potential fragmentation, loss of structural integrity, and plastic pollution if not removed or replaced over time. However, a specific description of project costs under site-specific conditions is lacking in the area, except for a few reports, including [23], where a description of cost and logistic performance is reported, highlighting the need for adaptation strategies to increase productivity of different temporal and spatial project scales. Each organization should conduct additional studies to understand site-specific needs. Regarding the selection of coral species, branching morphologies such as *Acropora*, *Echinopora*, *Porites*, and *Pocillopora* are favored over others. The relatively low diversity of species and morphologies is likely related to the stocking and nursery techniques, which are best adapted to branching morphologies; in addition, asexual reproduction through fragmentation is relatively easy, cost-effective, and available for any member of the community. Although several genera were reported, the restored assemblages remain narrower than natural reef communities, suggesting functional diversity may still be limited. Thus, the combination of logistical ease and low cost likely drives the selection of coral species. Such a strategy, besides being adapted to local needs, might help mitigate negative effects such as monoculture of outplanted corals and low genetic and species diversity, which are both reported causes of failures in restoration projects [8,24].

The limited consideration of genetic diversity in restoration projects is an important concern. Many initiatives rely heavily on asexual propagation of branching corals such as Acropora, resulting in monocultures that may be less resilient to environmental stressors and diseases. While sexual reproduction techniques can play a key role in enhancing reef resilience and long-term success, there are also other practical ways to integrate genetic diversity. For example, sourcing fragments from multiple donor colonies across different reefs and habitats helps capture a broader range of genotypes [24], which may be a more realistic approach for many projects in the Western Indian Ocean.

#### 4.1.3. Community involvement and visibility of restoration efforts.

The online survey conducted as part of this study has identified at least 22 case studies, showing an increase in the last few years compared to the 9 reported in [8]. This change over time resulted from an increase in restoration sites in the WIO, as well as from the more similar research and contacts used in this study for this area. However, many sites and case studies may not be readily apparent due to a lack of proper visibility, whether peer-reviewed publications or online (e.g., websites). Despite being an area with a relatively low number of cases compared to the USA, the Philippines, Indonesia, and Thailand, the 22 case studies show increasing attention to restoration practice, even in remote areas such as Comoros and Mayotte. Our survey showed that restoration projects appeared to be mostly driven by community-based organizations (27%), with NGOs representing 35%. Of the 22 sites, 76% of respondents reported the involvement of the community at different levels (e.g., from pre-implementation to post-outplanting). Community-based restoration projects and strong of the local community engagement have been pivotal to the long-term success of restoration actions (Sebastien et al., 2024). However, gaps in technical and ecological knowledge, techniques, and restoration planning can hinder optimization of restoration projects, calling for a specific approach for projects with high levels of community involvement. Workshops, training, and the use of local language were proposed as tools to overcome such gaps and to be included as standard procedures in community-based projects [25].

#### 4.1.4. Project objectives and alignment with restoration outcomes.

Project objectives in the WIO vary from a general need to re-establish reef ecological function to a more specific increase of fish population and creation of tourism attractions. Addressing objectives is a key component of a successful restoration program, and lack of clearly defined objectives has been identified as one of the major causes of failures in restoration projects [8]. A lack of specificity or relevance to local needs has also been commonly criticized, highlighting the need for considerable analysis before a restoration project [26]. For example, the lack of specific objectives to increase socio-economic value is one of the gaps in most restoration projects, which are mostly focused on enhancing coral survival. However, it has been demonstrated that social and economic outcomes can influence the success or pressures applied to a reef [27]. Limitations in the definition of objectives should be addressed at the beginning of a project or strategically mitigated during an ongoing project to increase the chance of success [8].

#### 4.1.5. Monitoring, socio-economic integration, and definitions of success.

The survey also highlighted the imbalance in the baseline and project implementation monitoring. Most of the case studies (86%) reported collecting baseline information before starting restoration activities, but only 38% included socio-economic indicators, even though they indicated that goals and objectives related to these indicators were included. Socio-economic monitoring, most commonly includes indicators such as changes in household income linked to restoration activities, employment generation, stakeholder participation rates, tourism-related revenue, and community perceptions of reef condition. This overview suggests that failures to meet restoration goals could be associated with a lack of understanding of the baseline. In fact, without drawing a clear picture of the previous and current reef conditions, the drivers of degradation, and local socio-economic needs, restoration will fail in the first planning step [28]. The monitoring indicators used in the WIO have been reported to be related to corals and fish communities. However, restricting the information collected to only two of the several components of a reef ecosystem might hinder the ability to show improvements. At a minimum, restoration monitoring should include coral survival and growth, colony condition, fish biomass or abundance, and basic socio-economic indicators, following existing regional guidance, such as the study in [29].

Standardization of monitoring and meaningful community involvement emerged as closely interconnected rather than separate challenges. While the lack of harmonized protocols limits comparability and learning across projects, many community-led initiatives rely on informal practices due to limited access to training, manuals, and technical guidance. Regionally relevant resources such as Coral Reef Monitoring in Eastern Africa: A Guide for Communities [1] provide practical protocols tailored to community-based initiatives and could serve as a foundation for harmonizing ecological and socio-economic monitoring approaches across the Western Indian Ocean. Integrating regionally adapted restoration manuals with participatory training approaches could improve data consistency while reinforcing local ownership, thereby strengthening both ecological rigor and long-term social sustainability.

### 4.2. Success of coral restoration in the WIO

Emerging trends in coral reef restoration in the WIO region include notable successes through the integration of novel technologies and community-driven approaches that enhance ecological and socioeconomic outcomes. Autonomous Reef Monitoring Structures (ARMS) have been applied in Madagascar, recognized for their dual role in monitoring biodiversity and contributing to habitat complexity, thus fostering more resilient ecosystems [30–32]. These modern ARMS differ from earlier Artificial Reef Matrix Structures [33] by incorporating high-throughput metabarcoding, offering detailed insights into cryptic species and ecosystem changes that inform restoration strategies.

Advancements in artificial reef designs using eco-friendly materials, such as biogenic structures, 3D-printed modules, or limestone, continue to improve habitat suitability and restoration success, although more recent studies could further validate these innovations [34]. The integration of participatory strategies places local communities at the center of conservation efforts, ensuring restored reefs meet ecological goals while supporting livelihoods through fisheries and ecotourism.

Research and ecological knowledge of coral restoration are rapidly increasing in Kenya and Seychelles, where dedicated teams share lessons learned with a scientific approach, supporting other regional projects. In Kenya, this institutional progress is further reflected in the development of the draft National Coral Reef Restoration Protocol, which provides guidance on site selection, monitoring indicators, implementation standards, and national-level coordination mechanisms. Such frameworks represent an important step toward harmonizing methodologies and strengthening governance of restoration activities. Both Seychelles and Mauritius are advancing plans to incorporate sexual reproduction techniques, to boost restoration success, capacity-building, and knowledge-sharing.

In 2018, Nature Seychelles released the first Coral Restoration Toolkit [35], an open-access resource that compiles nearly eight years of experience in site/species selection, nursery construction, maintenance, and outplanting techniques. Successes in coral survival, increased fish populations, and improved livelihoods have been reported across several countries, including Comoros, Kenya, Madagascar, Mauritius, Mayotte, Seychelles, and Tanzania. The human dimension remains pivotal for long-term restoration success [28,36,37], with strong links between coral restoration and communities throughout the WIO despite some scientific gaps.

The lessons learned highlight the need to communicate both successes and failures transparently, promoting collaboration and preventing duplication of efforts and methods across the region [8,11].

### 4.3. Challenges

Respondents highlighted several challenges during the in-person workshop, including a lack of funding, training, technical capacity, and institutional support. Adequate resources and sustained funding are essential to maintain interventions over larger spatial and temporal scales. Short-term projects with limited funding cycles are prone to failure (8).

Climate change remains a significant challenge. Notably, only three projects explicitly identified climate change as a direct constraint. This comparatively low reporting frequency should be interpreted cautiously and does not necessarily reflect low climate vulnerability in the region. First, the survey predates the global 2024 mass bleaching event, which affected large areas of the WIO and may significantly alter restoration trajectories. Second, many of the documented projects are relatively recent and short-term, and may not yet have experienced severe marine heatwaves within their monitoring period. As a result, climate-related impacts are likely underreported rather than absent. Third, most projects are relatively short-term and may not yet have experienced a severe bleaching episode within their monitoring period.

The WIO hosts approximately 16% of the world’s coral reefs and is considered to harbor the second highest peak of coral reef biodiversity globally, after the Coral Triangle (1). Recent marine heatwaves reinforce the need to evaluate restoration performance under repeated thermal stress rather than under average conditions alone. Future assessments should therefore explicitly examine restoration outcomes under extreme thermal events to determine whether current species selection, genetic sourcing strategies, and attachment methods confer meaningful resilience under projected climate scenarios.

A critical but often overlooked challenge is achieving full buy-in from subsistence fishing communities for restoration and management areas. Sustainable restoration efforts depend heavily on the support and cooperation of local fishers, who play a pivotal role in reef stewardship and compliance with management plans. Without their engagement, restoration sites risk overexploitation or damage, undermining ecological and social outcomes.

Knowledge gaps remain significant barriers. Many organizations willing to engage in coral restoration lack essential ecological and technical skills. Empowering practitioners and communities through targeted training in socio-ecological monitoring, scientific data collection, and advanced scuba skills will enable more effective and sustained restoration efforts. Project management skills are also often lacking; around 12% of respondents reported deficiencies here,resulting in projects that fail to align achievements with stated goals [8,38]. These capacity constraints are reflected in inconsistent monitoring and reporting practices across projects. Among the 22 identified initiatives, fewer than half reported monitoring coral survival beyond the first year, and only a small subset documented outplant numbers in a standardized manner. Metrics related to restored area, donor-site condition, or longer-term ecological change were rarely reported. Such limitations restrict the ability to evaluate ecological effectiveness, compare outcomes across sites, or assess cumulative regional impact. Based on these challenges, priority actions include: (1) establishing regionally harmonized monitoring indicators; (2) strengthening training and capacity building for community-led projects; (3) diversifying species and genotypes used in restoration; and (4) aligning project timelines with long-term funding and climate risk.

To maximize long-term success, restoration efforts must clearly define goals and objectives early in the planning stages. Applying the SMART framework (Specific, Measurable, Achievable, Relevant, and Time-bound) can help guide project design, resource allocation, and outcome evaluation [39].

## 5.0. Conclusion

This study provides the most comprehensive overview to date of coral reef restoration activities across the Western Indian Ocean (WIO). The questionnaire and workshop revealed 22 active restoration projects spanning eight countries, employing diverse techniques including direct transplantation, coral gardening, substrate stabilization, and innovative approaches such as Autonomous Reef Monitoring Structures (ARMS). Restoration efforts have primarily focused on fast-growing branching corals, particularly Acropora, and have shown positive ecological outcomes, including increased coral cover, greater fish diversity, and increased natural recruitment. Community involvement was reported in 76% of projects, contributing to ecological monitoring, outplanting, and awareness activities, and reinforcing socio-economic benefits such as alternative livelihoods and increased local stewardship. Despite these successes, significant challenges persist, including limited funding, insufficient technical and ecological capacity, lack of standardized monitoring, low genetic diversity of outplanted corals, and vulnerability to climate change and destructive fishing practices. Collectively, these findings underscore the importance of integrating ecological, technical, and social considerations to enhance the effectiveness and sustainability of restoration initiatives.

Addressing these challenges requires coordinated action at multiple levels. Based on the evidence from this study, we propose the establishment and operationalizing the WIO Coral Reef Restoration Network (WIOCRRN) to facilitate knowledge exchange, capacity building, and resource mobilization. Guided by the CARE framework – Capacity, Access, Research, and Enhancement – the network would aim to harmonize monitoring practices, strengthen practitioner skills, improve access to sustainable funding, and align restoration actions with both ecological resilience and community priorities.

The term “methodological and socio-economic enhancement” therefore refers to the combined actions of improving restoration techniques, standardizing monitoring, and strengthening community engagement and capacity to ensure measurable ecological and social outcomes.

Establishing a regional restoration network will help bridge gaps between science, policy, and practice, enabling adaptive management, sharing lessons learned, and preventing duplication of efforts. While the WIOCRRN aligns conceptually with broader regional initiatives such as the Great Blue Wall, which aims to restore coastal ecosystems, explicit WIO participation in this initiative should be verified before direct attribution. Regardless, a coordinated regional network is critical for scaling restoration efforts, ensuring ecological resilience, and supporting the communities that depend on coral reef ecosystems. For practitioners, future efforts should prioritize clear objective setting, minimum standardized monitoring indicators, diversification of species and donor sources, and stronger integration of socio-economic outcomes alongside ecological metrics. For policymakers and funding institutions, enabling long-term restoration success will require sustained financing mechanisms, policy support for community-led initiatives, and regional coordination to reduce duplication and enhance learning across projects.

## Supporting information

S1 Appendix Survey Questionnaire on Coral Reef Restoration in the WIO. (DOCX)

S1 Table List of coral reef restoration initiatives in the Western Indian Ocean (WIO) with corresponding locations and GPS coordinates. (DOCX)

S2 Table Coral reef restoration projects and their locations in the Western Indian Ocean region. (DOCX)

S3 Table Forms of community engagement reported by coral reef restoration initiatives in the Western Indian Ocean. Numbers indicate the count of projects reporting each engagement type.
(DOCX)

S4 Table Key lessons learnt from coral reef restoration practitioners across the WIO. (DOCX)

S1 Fig Number of peer-reviewed publications on coral reef restoration in the Western Indian Ocean per year, based on the literature reviewed in this study. (DOCX)
