# Supplementary material for: A review of coral reef restoration initiatives in the Western Indian Ocean Region
Source: PLoS One. 2026 May 8;21(5):e0348015. doi: 10.1371/journal.pone.0348015 (PMC13155574; doi:10.1371/journal.pone.0348015)
Supplement: S1 Appendix — (DOCX) [file pone.0348015.s001.docx]

Appendix 1: Survey questionnaire on coral reef restoration in the WIO with accompanying link to the form

**Purpose:**

This survey aimed to document the status, techniques, and lessons learned from coral reef restoration initiatives in the Western Indian Ocean (WIO) region. Participation was voluntary, and responses were anonymized.

**Section 1: Project Details (Mandatory)**

1. **Country where the project is based:** ________
2. **Name(s) of the project area(s):** ________
3. **Number of active restoration sites:** ________
   - Please provide GPS locations (latitude/longitude).
4. **Area actively restored (Ha):** ________

**Section 2: Organizational Information (Optional)**

1. **Leading organization(s) conducting the restoration project:** (Tick all that apply)
   - Government
   - Tourism operator
   - NGO
   - Research Institute/University
   - Private
   - Community-Based Organization (CBO)/Beach Management Unit (BMU)
   - Other: ________
2. **Stakeholders directly involved in planning and implementing the restoration project:** (Tick all that apply)
   - Tourism, fishing, or other businesses
   - Local government agencies
   - Marine park managers
   - National government
   - NGOs
   - Tourism operators
   - Research Institutes
   - Private organizations
   - Community organizations/BMU/CBO
   - Citizen volunteers
   - The lead organization conducted all aspects
   - Other: ________

**Section 3: Goals and Achievements**

1. **Goals of the restoration project:** (Tick all that apply)
   - Speed up reef recovery following a disturbance
   - Reestablish a self-sustaining, functioning reef ecosystem
   - Relocate corals before planned disturbance
   - Reduce pressure on existing reefs
   - Create tourist attractions
   - Provide opportunities for community/tourism involvement in conservation
   - Create new fish habitat
   - Scientific research
   - Other: ________
2. **Achievements linked to project goals:** ________

**Section 4: Data Collection and Techniques**

1. **Baseline ecological data collected?**
   - Yes
   - No
   - If yes, specify: ________
2. **Socio-economic data collected?**
   - Yes
   - No
3. **Coral reef restoration techniques used:** (Tick all that apply)
   - Direct coral transplantation
   - Substrate creation (e.g., artificial reefs)
   - Coral gardening (nursery + out-planting)
   - Micro-fragmentation
   - Substrate stabilization
   - Bio-rock
   - Other: ________
4. **Was restoration training conducted prior to implementation?**
   - Yes
   - No
5. **Coral types used:** (Tick all that apply)
   - Branching
   - Massive
   - Foliose/Plate
   - Encrusting
   - Other: ________
6. **Coral genera/species used:** ________
7. **Source of coral transplants:** (Tick all that apply)
   - Loose fragments (corals of opportunity)
   - Whole colonies from nearby locations (same reef)
   - Whole colonies from other locations
   - Fragments from nearby colonies (same reef)
   - Fragments from other locations
   - Self-sustaining nurseries
   - Sexually produced coral larvae
   - Did not transplant corals
   - Other: ________
8. **Method used for attachment (e.g., adhesive, cable ties):** ________

**Section 5: Monitoring and Community Engagement**

1. **Monitoring and maintenance activities:**
   - Duration (weeks/months/years/ongoing): ________
2. **Community involvement:**
   - Yes
   - No
   - If yes, describe: ________

**Section 6: Project Duration and Success Metrics**

1. **Duration of restoration project:** ________
2. **Short-term (3-year) and long-term (8-year) objectives:** ________
3. **Is the project successful?** (Only for projects >1 year old)
   - Yes
   - Partially
   - No
4. **Measurable indicators of success (ecological and social):** ________

**Section 7: Lessons Learned and Future Directions**

1. **Important lessons learned:** ________
2. **Challenges experienced and how they were addressed:** ________
3. **Emerging trends or technologies that may advance coral restoration:** ________
4. **Is a WIO coral reef practitioner network valuable?**
   - Yes
   - No
   - Maybe
5. **Objectives for establishing a WIO network:** ________
6. **Interest in joining the network:**
   - Yes
   - No
   - Maybe
7. **Permission to be contacted for further information:**
   - Yes
   - No
   - Maybe

Link to Questionnaire: <https://forms.gle/dJRMoGan73R5rozS6>
