# Supplementary material for: A review of coral reef restoration initiatives in the Western Indian Ocean Region
Source: PLoS One. 2026 May 8;21(5):e0348015. doi: 10.1371/journal.pone.0348015 (PMC13155574; doi:10.1371/journal.pone.0348015)
Supplement: S1 Table — (DOCX) [file pone.0348015.s002.docx]

Table S1**. List of coral reef restoration initiatives in the Western Indian Ocean (WIO) with corresponding locations and GPS coordinates**

| **Project Name** | **Latitude** | **Longitude** |
| --- | --- | --- |
| Reef Rescuers | -4.32750 | 55.65722 |
| Reef Rescuers | -4.33275 | 55.65500 |
| Reef Rescuers | -4.33530 | 55.65750 |
| Reef Rescuers | -4.33417 | 55.66611 |
| Reef Rescuers | -4.33536 | 55.65990 |
| Coral Restoration Mauritius | -20.30109 | 57.35701 |
| Coral Restoration Mauritius | -20.30003 | 57.36069 |
| Coral Restoration Mauritius | -20.33552 | 57.79167 |
| Coral Restoration Mauritius | -20.33920 | 57.79332 |
| Coral Restoration Mauritius | -20.45347 | 57.70051 |
| Coral Restoration Mauritius | -20.43452 | 57.74192 |
| Coral Restoration Mauritius | -20.14389 | 57.48411 |
| Coral Restoration Mauritius | -20.02836 | 57.54287 |
| Coral Restoration Mauritius | -20.03553 | 57.53373 |
| Coral Reef Restoration-Fisi Somanga | -8.39417 | 39.35361 |
| Coral Reef Restoration | -7.40083 | 39.54639 |
| Coral Reef Restoration | -6.44472 | 38.90000 |
| Coral Reef Restoration | -5.82000 | 39.38333 |
| Matumbawe Hai | -7.13333 | 39.52250 |
| ARMS Restore | -23.09889 | 43.48583 |
| CORES | -12.97694 | 40.51694 |
| Tuliary | -11.37583 | 43.31444 |
| REEFolution Trust | -4.65033 | 39.38697 |
| REEFolution Trust | -4.65804 | 39.38052 |
| REEFolution Trust | -4.65949 | 39.38607 |
| REEFolution Trust | -4.65963 | 39.39023 |
| REEFolution Trust | -4.71505 | 39.37124 |
| REEFODIVERS | -4.29333 | 39.59537 |
| Wasini BMU restoration project | -4.66666 | 39.38333 |
| KICOWA | -1.96431 | 41.30624 |
| PMCCC | -2.18303 | 41.03975 |
| THE OCEAN TRUST | -2.17528 | 41.09472 |
| Oceans Alive Trust | -3.80361 | 39.83611 |
