# Supplementary material for: A review of coral reef restoration initiatives in the Western Indian Ocean Region
Source: PLoS One. 2026 May 8;21(5):e0348015. doi: 10.1371/journal.pone.0348015 (PMC13155574; doi:10.1371/journal.pone.0348015)
Supplement: S2 Table — (DOCX) [file pone.0348015.s003.docx]

Table S2. Coral reef restoration projects and their locations in the Western Indian Ocean region.

| Project | Location |
| --- | --- |
| 1. ARMS Restore | Madagascar |
| 1. Pointe-aux-Feuilles | Mauritius |
| 1. La Cambuse | Mauritius |
| 1. Pointe D’Esny | Mauritius |
| 1. Coral Reef Restoration (mainland Tanzania) | Tanzania |
| 1. Coral Reef Restoration – Fisi Sonanga | Tanzania |
| 1. CORES | Tanzania |
| 1. KICOWA | Kenyan coast |
| 1. Matumbawe Hai | Tanzania |
| 1. Oceans Alive Trust | Kenya |
| 1. PMCC | Seychelles |
| 1. Reef Rescuers | Seychelles |
| 1. REEFDIVERS | Mozambique |
| 1. REEFolution Trust | Kenya |
| 1. The Ocean Trust | South-west Madagascar coast |
| 1. Wasini BMU Restoration Project | Kenya |
| 1. Utopian Project | Reunion |
| 1. So Naturel Project | Reunion |
| 1. Corecif Project | Reunion |
