# Supplementary material for: A review of coral reef restoration initiatives in the Western Indian Ocean Region
Source: PLoS One. 2026 May 8;21(5):e0348015. doi: 10.1371/journal.pone.0348015 (PMC13155574; doi:10.1371/journal.pone.0348015)
Supplement: S3 Table — Numbers indicate the count of projects reporting each engagement type. (DOCX) [file pone.0348015.s004.docx]

Table S3. Forms of community engagement reported by coral reef restoration initiatives in the Western Indian Ocean. Numbers indicate the count of initiatives reporting each type of engagement.

| **Types and levels of local community engagement in restoration activities** |
| --- |
| 1. Participation in the entire process: training, site selection, artificial reef implementation, and ARMS deployment. |
| 1. Involvement from nursery maintenance through transplantation and monitoring. |
| 1. Engagement in pre-implementation, implementation, maintenance, and monitoring stages. |
| 1. Community-led projects with involvement in every stage, including consultation workshops to build consensus. Monitoring and all other activities involve the community. |
| 1. Through the establishment of the community closure, the pioneer members of coral restoration are members of the KCW-CBO. Community ownership promoted via community closures; fishers mobilized as trainees in restoration programs. |
| 1. Sustained involvement lasting at least two years. |
| 1. Participation in coral cutting, allocation to structures, and area surveying to prevent damage. |
| 1. Involvement of fishermen in restoration activities. |
| 1. Mkwiro BMU and REEFolution have worked closely together from the start of the project, starting with a collaborative grant, project plan and launch of a community managed area in which the restoration could take place. REEFolution is currently employing local people. |
| 1. Training of two local fishers and four marine graduates, including SCUBA certification and online courses.by The Nature Conservancy and the Reef Resilience Network. |
| 1. Involvement in education, awareness, baseline surveys, artificial reef construction, coral fragment collection, out-planting, and maintenance. |
| 1. Participation of approximately 3,000 fishermen and 1,500 women in restoration efforts. |
| 1. Employment of community members to assist with tying coral fragments onto reef structures. |
| 1. Community participation across problem identification, planning, planting, and monitoring phases. |
| 1. Project inception at the community and planning stages. |
| 1. Engagement in training, planning, baseline surveys, and construction/deployment of artificial reefs. |
