# Supplementary material for: A review of coral reef restoration initiatives in the Western Indian Ocean Region
Source: PLoS One. 2026 May 8;21(5):e0348015. doi: 10.1371/journal.pone.0348015 (PMC13155574; doi:10.1371/journal.pone.0348015)
Supplement: S4 Table — (DOCX) [file pone.0348015.s005.docx]

Table S4. Key lessons learnt from coral reef restoration practitioners across the WIO.

| **Thematic area** | **Report from respondents on lessons learnt** |
| --- | --- |
| **1)** Climate Change | Climate change impacts are impossible to deal with using normal coral gardening |
|  | Coral bleaching is the main affecting factor resulting in mass mortality for coral recruits and juveniles, |
| **2)** Funding | Very large amounts of funding need to be mobilised |
|  | Coral restoration is expensive |
| **3)** Organizational | Only large institutional-level, rather than small community projects have a medium to long-term impact. |
|  | Forge strong partnerships with local organizations, government agencies, and scientific experts to leverage resources, expertise, and support for effective project implementation and sustainability |
|  | Transdisciplinarity, co-design, science-based and synergetic partnership (between the main stakeholders) are among the most important key factors |
|  | Proper monitoring and administration of the project will build trust and credibility for the organization |
|  | Often, it is only a few people in the right position (leaders) who can really help a project take off |
|  | Foster collaboration |
|  | Forge good relationships between KWS and the community |
|  | Partnership is key to successfully implementing the project |
|  | Stakeholder engagement is a must-do before the implementation of any project |
|  | Collaboration among key stakeholders is critical for the success of coral restoration |
| **4)** Governance | Government has to back the efforts 100% with more than words. |
| **5)** Community Involvement | Prioritize meaningful engagement with local communities from project inception to implementation by ensuring their active participation, ownership, and support throughout the process |
|  | Creation of alternative livelihoods is necessary for conservation to progress |
|  | Start with community engagement and the government will (hopefully) follow |
|  | Actively involve community members in every step |
|  | Community can play a major role towards the restoration of corals through both active and passive regeneration |
|  | Community ownership and stewardship are important |
|  | Ownership of restoration projects by the community is possible when they are involved from the beginning |
|  | Increasing the capacity of the community to lead reef restoration projects is an important lesson. Scuba diving, monitoring |
|  | Ownership of the project has led its the success |
|  | Community buy-in is essential for the success of the project |
|  | Inclusion of women and youth gives the project more visibility |
| **6)** Threats | Destructive fishing practice |
| **7)** Duration to realise benefits | We need to be patient with coral restoration |
|  | Benefits are long-term |
| **8)** Monitoring | Carry out regular monitoring to remove algae and other harmful elements |
|  | Establishing robust monitoring and evaluation mechanisms to track project progress, assess outcomes, and adapt strategies as needed to maximize impact and success. |
| **9)** Awareness and Capacity Building | Prioritizing education and awareness programs to foster understanding, appreciation, and stewardship of natural resources among local communities, promoting long-term conservation efforts and sustainability |
|  | Restoration is an effective way to create awareness, both locally and internationally |
| **10)** Management | It is better to protect the reef than to plant |
|  | Combine restoration with conservation (e.g. locally managed areas) |
|  | Scientific input is paramount to coral restoration |
| **11)** Ecological Considerations | Use a diversity of corals (different species withstand different stressors) and strong coral attachment is important for coral survival. Don’t outplant corals during heat stress (March, April) or before strong monsoon winds. |
|  | Site selection is important in restoration |
|  | That a degraded area can be restored using various techniques |
|  | The importance of good site selection, and matching the most appropriate restoration method to the conditions, importance of managing corals out of the water and how to conduct all parts of the process to minimise mortality of coral fragments |
|  | Protecting marine ecosystems offers room to improve on species and ecosystem integrity |
