# Supplementary material for: A review of coral reef restoration initiatives in the Western Indian Ocean Region
Source: PLoS One. 2026 May 8;21(5):e0348015. doi: 10.1371/journal.pone.0348015 (PMC13155574; doi:10.1371/journal.pone.0348015)
Supplement: S1 Fig — (DOCX) [file pone.0348015.s006.docx]

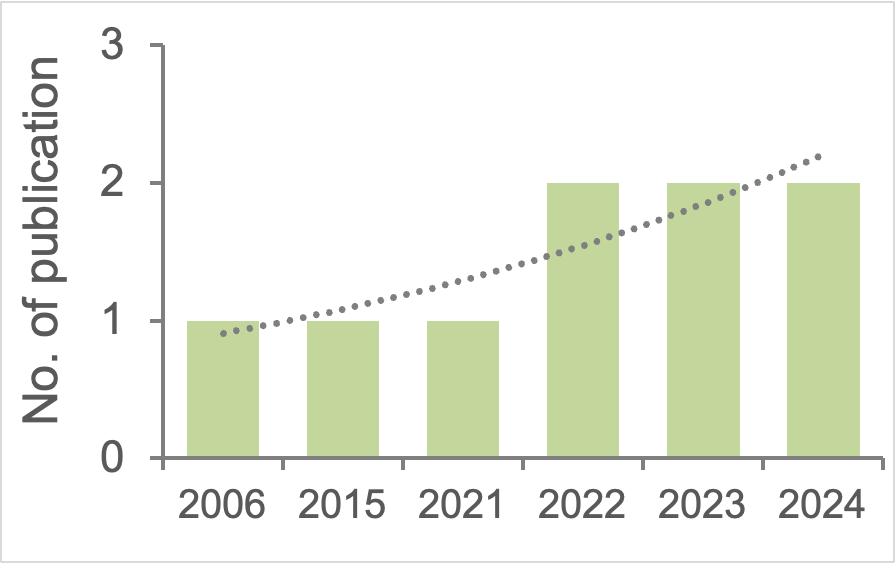


S1 Figure. Number of peer-reviewed publications on coral reef restoration in the Western Indian Ocean per year, based on the literature reviewed in this study.
